# Exotic Plants Used by the Hmong in Thailand

**DOI:** 10.3390/plants8110500

**Published:** 2019-11-14

**Authors:** Varangrat Nguanchoo, Prasit Wangpakapattanawong, Henrik Balslev, Angkhana Inta

**Affiliations:** 1 Department of Biology, Faculty of Science, Chiang Mai University, Chiang Mai 50200, Thailand; Taeyya@hotmail.com (V.N.); prasitwang@yahoo.com (P.W.); 2 Department of Biological Sciences, Aarhus University, DK-8000 Aarhus, Denmark; henrik.balslev@bios.au.dk; 3 Center of Excellence in Bioresources for Agriculture, Industry and Medicine, Chiang Mai University, Chiang Mai 50200, Thailand

**Keywords:** acquisition, adaptation, immigration, ethnobotany, non-native plants, traditional knowledge, transmission

## Abstract

Exotic species are an integral part of the plants used by many ethnic groups, but they usually receive little attention and have been considered alien to the ethnobotanical data. Here, we analyze the plants used by Thai Hmong refugees that are not native to their current habitats in Thailand. We attempt to understand the sources of this knowledge. Do people maintain the original traditional knowledge related to exotic species when they migrate to a new region, or does new knowledge originate from acculturation? We interviewed 16 specialist Hmong informants in Nan province, Thailand, about their traditional knowledge of 69 exotic species used. Acquisition of this knowledge has a long history; several species are the same as plants used by the Hmong in China and other countries, others are globally useful species which have become part of the pool of species that the Hmong have developed local knowledge about. However, migration also involves the integration of local knowledge from other cultures, and also adapts them to function in urban settings. This includes using closely related exotic taxa that replace some of the species they used in their original homelands. The migrants’ traditional knowledge in their new habitats is more complicated and also involves the development of local knowledge that is entirely new.

## 1. Introduction

Exotic species are such ones that have been introduced by humans into new places outside their native habitats [1]. Some exotic plants have long histories of introduction in cultivation, and they may reach a stage where they produce offspring without intervention by humans and, finally, they may become naturalized [2,3,4]. For instance, *Solanum torvum* Sw. was recently found for the first time in Europe as naturalized in a riverbed, and it probably escaped from cultivation because it is used as rootstock for some edible Solanaceae species (e.g., *S. lycopersicum* L., *S. melongena* L.) [5]. Some exotic plants have potential to spread rapidly and to become more competitive than local plants [6,7]. They are then sometimes characterized as weeds or invasive species that have negative effects on the biodiversity [6,7,8]. Weeds and invasive species are unwanted plants from the human point of view [1,4] because in farmlands, they may reduce agricultural crop yields when they compete with the cultivated plants for light, nutrients, and other resources [9].

Ethnobotanists have mostly focused on native plants [10] that evolved within the study regions where they have their natural interactions with the environment and other organisms. Exotic species may also be present in the same regions, but they have usually received less attention than the native plants [10]. As use of exotic species generates new knowledge [11], replacement of native plants by exotic ones can threaten the traditional system [12]. However, the exclusion of exotic plants is unfortunate for ethnobotanical studies because ethnobotany should include the study of all interactions of plants and people through utilization. Ethnobotany combines several disciplines and aims at understanding the close connection between plants and human societies [13]. Exotic plants are many times closely associated with human activities because they are introduced into new areas for their utilization [14]. Some plants have long histories of being moved around with people and a fundamental concept in ethnobotany is to tell the history of plants in a particular area [15,16]. Nowadays, many exotic plants are used for food, medicine, horticultural and other ecosystem services, including aesthetic enjoyment [17]. Therefore, exotic plants play an integral role and it is appropriate to study them in ethnobotanical contexts.

Not only do urban societies use exotic plants, but also ethnic groups who live under very rural conditions commonly use exotics as food and medicinal herbs [18,19,20]. Sometimes the use is so intensive that they become part of the group’s cultural identity because these exotic plants are widespread and easy to access in their community or home gardens [21]. In South Africa, 34 exotic plant species were used to treat health conditions by local healers among four major ethnic groups: Xhosa, Zulus, Vendas, and Swatis [22]. Another study from South Africa documented 300 exotic plants that are used as medicinal herbs and traded in local markets [23]. In India, 24 exotic plants were used as medicine by tribal groups of Sonaghati [17]. However, in Southeast Asia, it appears that exotic species are less commonly mentioned in the ethnobotanical literature, possibly because they are less commonly used.

Refugees are people who have fled their home country, and who are unable to return to their country because of persecution, war, or political opinion [24]. Modification of knowledge has appeared among refugees because of exposure to different biological and cultural influences [25]. Acculturation and adaptation to appropriate urban life are common during resettlement and increase proportionally to the length of residence [26]. Therefore, use of exotic plants among immigrants is influenced by both their culture of origin that they bring with them and the culture from their new host culture that causes the acculturation process [27]. For instance, the knowledge of Krummhübel herbalists in Poland were transmitted from two Protestant refugees from Prague [28]. Eight exotic plants were frequently used in several herbal recipes. From the overlap between names of exotic and old herbs, they assumed these uses could refer to the origin of their knowledge from the monastic tradition since the 16th century.

The Hmong are one of several ethnic minority groups in Southeast Asia. They migrated from southwestern China as far south as Vietnam, Laos, Burma, and Thailand during the 18th and 19th centuries because of war in their homelands [29]. They have also sought refuge in the United States and Europe [30]. They crossed the Mekong River from Laos and dispersed to various parts of northern Thailand [31]. The number of Hmong in Thailand increased rapidly after the end of the Indochina Wars in 1954. Currently, the Hmong population is the second largest ethnic minority in Thailand [32]. Their traditional botanical knowledge has been passed down since ancient times through oral tradition [33,34]. Several plants from the forest and Hmong home gardens are used in daily life, ceremonies, and rituals, and these uses have been reported in several ethnobotanical studies [33,34,35,36,37,38,39,40,41,42]. In these publications, exotic species are only briefly discussed compared to the native species, despite the fact that they may also serve as important plants. For example, 60 exotic plants were used by Hmong in Chiang Mai, northern Thailand [37]. Most of these plants (70%) were found in their home gardens [37] where species are easier to find compared to in the forest because they are cultivated and protected [43]. Laotian Hmong who migrated to California, cultivated both Asian native plants and common vegetables of which seeds were purchased in American stores or obtained from their American neighbors [44]. Ethnobotanical data about these non-native useful plants is lacking and should be seriously studied, especially in the refugee context. Ethnic migration will give rise to uncertainty about the origin of knowledge about exotic plants. This research has the objectives of (1) to compile information about exotic plants used by the Hmong, (2) to determine the geographic origins of such plants, and (3) to determine the sources of knowledge about exotic plants among the Hmong, distinguishing between the traditional knowledge originating in their homelands before migration, and any new knowledge acquired in post-migration acculturation through contacts with different cultures and new ecological conditions.

## 2. Results

### 2.1. Exotic Species Used

A total of 69 exotic species in 62 genera and 37 families were recorded in the six Hmong villages (Table S1). The families with most species were Asteraceae (10%), followed by Amaranthaceae and Solanaceae (each with 7%), and Euphorbiaceae (6%) (Table 1).

Eight of the species were cosmopolitan weeds that occur in agricultural and forestry areas (Table 2). Even if these species are unwanted in the fields, they are at the same time used for many purposes by the Hmong. For instance, they use the leaves of *Chromolaena odorata* (L.) R.M.King & H.Rob. that invades crop fields and edges of forests to stop bleeding when there is an emergency in the field. The Hmong wear a triangle-shaped amulet pouch with *Mimosa pudica* L. fragments around their neck or pin it to their clothes to ward off evil spirits. *Celosia argentea* L. is an important medicinal weed used by the Hmong to treat amenorrhea and dysmenorrhea. Moreover, some weeds rapidly spread around and serve as animal feed such as *Amaranthus spinosus* L. and *Crassocephalum crepidioides* (Benth.) S.Moore. It is a good way to balance the weeds by natural control.

Of the 69 exotic species encountered, 58 were used as medicine, whereas smaller numbers were used for food, social purposes, animal feed, and materials (Table S1).

The medicinal exotic species were used by the Hmong to treat health conditions in 17 different categories. Most medicinal plants were used to treat diseases of the genitourinary system (17%), especially in menstrual cycle bleeding disorders, diseases of the digestive system (16%), and endocrine, nutritional or metabolic diseases, especially in nutritional disorders (11%). The menstrual cycle bleeding disorders included amenorrhea, dysmenorrhea, and female fertility. The popular species used in this category often had red and orange colors (e.g., *Celosia argentea*, *Impatiens balsamina* L., and *Tagetes erecta* L.). Digestive system disorders such as diarrhea, peptic ulcer, and stomachache are common. *Psidium guajava* L. was commonly used for treating diarrhea. The common methods for plant preparations are decoction (40%), cooking (22%), and pounding (18%). The plant materials are chopped and boiled for a prolonged period in water for oral consumption as an herbal tea. Culinary medicine is important to the Hmong who cook herbs in their meals. Clear chicken soup is a popular Hmong recipe using the removed entrails of black chicken that are boiled with a variety of herbs, and then salted to give the dish a mild taste. The soup is served with hot steamed rice. Exotic ingredients such as *Artemisia lactiflora* Wall. ex DC. and *Iresine herbstii* Hook. may be included showing the importance of exotic plants in this signature dish of the Hmong’s culture.

Exotic food plants were vegetables (53%), fruits (30%), carbohydrate sources (10%), and food additives (7%). Most of the exotic food plants were ones that are commonly eaten in Asia or worldwide (e.g., *Manihot esculenta* Crantz and *Zea mays* L.)

The traditional Hmong religion is animist, which often involves the protection against black magic and treatment of the negative effects of that magic, evil spirits and other bad things, such as soul-calling. The plants used for these purposes were often such ones that are valued as ornamentals in Thailand and elsewhere in the tropics. *Zephyranthes carinata* Herb. leaves were used to protect the owner from evil spirits by keeping it in a triangle-shaped amulet pouch. *Hippeastrum* × *johnsonii* was used to protect against evil spirits and *Gladiolus* × *hortulanus* L.H. Bailey was used in soul-calling rituals to treat the frightened soul when it had fallen away and was scared or panicked. *Caladium bicolor* (Aiton) Vent. is widely used as ornamental foliage plants because of many color patterns and variations. It is commonly used in the villages to treat pain from evil black magic. We are not aware of previous reports of the plant being used for this purpose.

### 2.2. Geographic Origins

**Figure 1:**
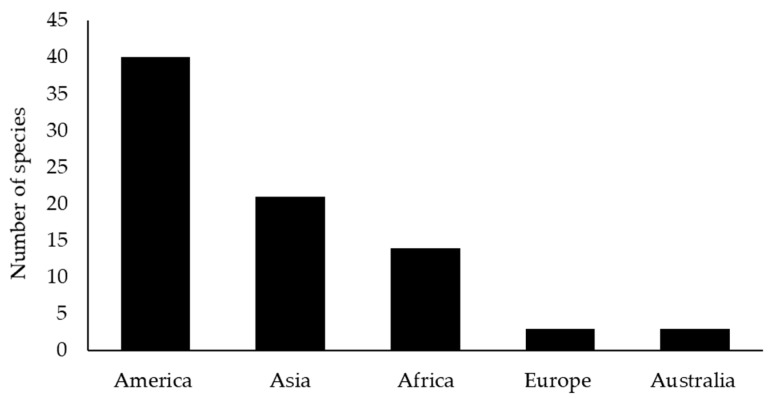
Geographic origin of 69 exotic plant species used by the Hmong in six villages in Nan province, Thailand.

The 69 species of exotic plants used in the seven Hmong villages originated from five regions (Figure 1). Most species (40) came from America and of these six species were weeds (Table 2). The second most important region of origin of the exotic species was Asia which had 21 species, of which 16 were native to China. For Chinese herbs, there was a consensus about their uses among the Hmong. For instance, *Artemisia lactiflora* and *Chrysanthemum indicum* L. were used as a tonic. *Artemisia vulgaris* L. was used to treat malaria and fever, and *Sedum sarmentosum* Bunge was used as a tonic and food for pregnant women. Fourteen species were native to Africa and only a few of the Hmong exotic species came from Europe and Australia.

### 2.3. Sources of Hmong’s Knowledge

Hmong’s knowledge of exotic plants is derived from four main sources. Most knowledge of exotic species (46%) was acquired from worldwide sharing. Many species were well-known and globally used the same as they were used for by the Hmong. Among the American species, the Hmong used *Manihot esculenta*, *Solanum lycopersicum*, and *Zea mays* for food and as cash crops, *Nicotiana tabacum* L. for smoking, and *Annona squamosa* L., *Carica papaya* L., and *Psidium guajava* as edible fruits. Of those native to Asia, many had been promoted during a long time of cultivation as directly edible or for commercial use. This was true for *Anethum graveolens* L., *Artocarpus heterophyllus* Lam*., Citrus maxima* (Burm.) Merr., *Psophocarpus tetragonolobus* (L.) DC., *Solanum melongena,* and *Solanum torvum.* Exotics that are native to Africa included *Aloe vera* (L.) Burm.f., which was commonly used to treat burns, *Crassocephalum crepidioides*, which was used for food, and *Lagenaria siceraria* (Molina) Standl., which was used for making utensils. Moreover, some exotic species are commonly used elsewhere in Thailand such as *Amaranthus spinosus* L., *Ayapana triplinervis* (Vahl) R.M.King & H.Rob., and *Passiflora foetida* L., which were eaten as local vegetables.

The second source of exotic plant knowledge was transmission from their original homeland to their new homeland (22%). Several species of plants used by the Hmong are the same as in Thailand, China and other countries where they have settled. Among these, *Impatiens balsamina*, *Iresine herbstii*, and *Mirabilis jalapa* L. were commonly used to treat menstruation disorders. *Tradescantia zebrina* Bosse was boiled in chicken soup as a tonic, and *Bryophyllum pinnatum* (Lam.) Oken was also used as a tonic, to treat muscular-skeletal disorders and injuries.

Interestingly, some of the uses of exotic plants appeared to have evolved on site and to be unique to the Thai Hmong and were not known in Hmong communities in other countries (19%). *Euphorbia tithymaloides* L. was used as a lactation stimulant. *Crinum × amabile* Donn ex Ker Gawl. was used to treat abscesses, bone fractures, bruises, and sprains (strains). *Verbena officinalis* L. was used to treat foot dermatitis and itchy rashes, which are common health conditions because the Hmong’s main occupation is as farmers and their feet are always in touch with soils and water.

Finally, the Thai Hmong had acquired new exotic plant knowledge (13%) from new habitats. These species were the first records of the plants being used by the Hmong, e.g., *Gladiolus* × *hortulanus*, which was used for social purposes and *Plectranthus scutellarioides* (L.) R.Br., which was used to treat flatulence and liver disorders.

## 3. Discussion

### 3.1. Exotic Species Used

As shown above, exotic plants are important in the Hmong culture. This has been documented in previous ethnobotanical studies that reported anywhere from 22–38% of the plants used by the Thai Hmong as being exotic [34,35,37,39,40,41,42,45]. The family with the most exotic plants used by the Hmong was Asteraceae. It is one of the largest plant families globally and is well-known for having many weedy species [9]. Asteraceae are often widely distributed because of their numerously light seeds, and they are dispersed by wind and adhesion [46]. Basically, weeds are plants that grow in places where they are not appreciated, and they are undesirably seen from a human point of view [4]. Here, we focus on the useful aspects of exotic weeds. We suggest that they should not be subjected to inattentive and indiscriminate eradication without consideration of their potential usefulness [4]. Good management of exotic weeds can be better achieved through accurate control than through complete elimination.

As for the medicinal plants, many exotic herbs were used to treat health conditions of women. Women’s healthcare is crucial to human life in most cultures, including the Hmong in Thailand who use a large diversity of herbs as remedies for such purposes [47]. Plants with reddish color are believed to be efficient in the treatment of blood-related ailments; therefore, red plants are often used to treat health problems connected to menstruation [48]. Nguanchoo (2014) found that the Hmong used many exotic species to treat common medical problems, for instance, nutritional and gastrointestinal disorders. When preparing the medicine, herbs were usually decocted, which is a simple pharmaceutical method and maybe the most common one for preparation of medicine since ancient times [49,50,51]. As in China, food plants and medicinal plants are mixed in diets and form an important link between food and health. It is very popular among the Hmong to boil herbs with chicken to make a clear soup. The soup was used as a tonic, which is an important part of the Hmong identity [52]. Tonic is commonly used in many cultures for balancing elements, enhancing immunity, and boosting rapid recovery [49].

For social purposes, exotic plants are part of Hmong beliefs and rituals that are deeply rooted in their culture. Therefore, we expected that the plants used by the Hmong for religious and ritual uses would be native plants that had been available to them forever. We did not expect to find exotic species in this category of uses. Exotic species would have been added to their plant arsenal long after their beliefs and rituals developed. Nevertheless, we found nine exotic species among those used for purposes related to beliefs and rituals. The uses of some of these were the results of acculturation because they are hybrids and newly introduced ornamental plants such as *Gladiolus × hortulanus* and *Hippeastrum × johnsonii.* However, some species have a long history such as *Zephyranthes carinata*, which is a sacred plant. A closely related species, *Z. rosea* is also used by the Hmong in Thailand [35,42,45] and in Minnesota, USA [53] and was brought with the migration from southern China becoming naturalized in Thailand [34]. Another exotic plant used in rituals is *Caladium bicolor*, which contains calcium oxalate crystal that may cause skin irritation, vomiting and be toxic when ingested [54]. However, calcium oxalate can be destroyed by drying or heating [55], so the Hmong pound the rhizome to make a liniment for areas that suffer pain from evil black magic. It produces intensive burning and itching that is strong enough to eliminate the evil power. This belief makes it possible to fight pain from magic with pain from poisoning. The oldest Amerindian charm, *Caladium bicolor*, is represented by a collection from 1837 in the Leiden herbarium. It is widely used by all 11 indigenous groups in the Guianas [56]. Organs of desired game animals were burnt into powder, mixed with the juice of the *Caladium* tuber and rubbed on the hunter’s body as a hunting charm. This had to be painful because the hunter had to be prepared for the pain and danger in the forthcoming hunt [56,57]. *Sansevieria trifasciata* Prain is commonly known as “snake plant”. The Hmong used it for protecting them from snakes and evil spirits and also to treat snake bites with a leaf poultice or decoction [37,45]. In Bangladesh, Kenya, India, and Nigeria, *S. trifasciata* is also used to repel snakes and to treat snake bites [58,59,60,61]. Supposedly, snakes do not like the appearance of the plant because of its shape and sharp margins of its leaves [58]. This supports the Doctrine of Signature, in which the physical characteristics of plants reveal their therapeutic value [48]. This led to the discovery of many medicinal plants and plant characteristics believed to be linked with a charm, ritual, or sacred plant [56]. It has been shown that the ethanolic extract of *S. trifasciata* induces potent antiallergic and anti-anaphylactic activity [62]. As mentioned above, many researchers revealed the use of at least 19 exotic species for ritual and belief-related uses in the Hmong culture [37,45]. Some of the previous reports agree with those recorded here, for example, that *Jatropha gossypiifolia* L. and *Sansevieria trifasciata* are grown around the Hmong’s houses to protect from evil spirits and to expel snakes, respectively. *Mimosa pudica* is kept in a triangular pouch as a sacred plant.

### 3.2. Geographic Origins

The Hmong used many plants introduced from America. This may not be surprising because native American plants became popular and widespread throughout the world following the exploration and colonization era, especially the voyages of Christopher Columbus and his discovery of the New World in 1492. Moreover, historical evidence shows that some plants were introduced into Asia in pre-Columbian times including *Zea mays* [63]. Many seeds were carried to be planted in Europe and subsequently rapidly spread to Asia and Africa where there were European colonies [64,65]. The same is true for the fruits and vegetables. They were introduced to European colonies along commercial sailing routes around the 16th century and have been cultivated there ever since. Examples include *Annona squamosa*, first cultivated in Malaysia by the Dutch [66], *Carica papaya* which was introduced to the Philippines by the Spanish [67], and *Cucurbita moschata* Duchesne, which was introduced to India, Southeast Asia, and Japan by the Spanish [68]. *Eryngium foetidum* L. was introduced in Southeast Asia by the Chinese using it as a substitute for coriander [69], and *Muntingia calabura* L. was probably first introduced in Thailand or Vietnam by the Portuguese [66].

The Hmong in our study also used many exotic herbs introduced from China. They frequently carried plants and seeds to be grown in their new homelands where they settled [33,34]. These helped in achieving endurance of their traditional knowledge and the transmission of it to new generations in the settled area. *Artemisia lactiflora* and *A. vulgaris* are native to China and both are important in traditional Chinese medicine for menstrual problems and liver disorders [70,71]. Leaves of *A. lactiflora* contain volatile oil and the Hmong in Thailand boiled it in chicken soup as a tonic [34,40,41,45,52]. *Artemisia lactiflora* was reported for Thailand in the scientific literature for the first time in 1993. It was documented that it had been brought from southern China with the Hmong migration [34]. *Artemisia vulgaris* has for long been used to treat malaria and as a fever remedy by the Hmong in Thailand and Minnesota [53]. Moreover, many exotic plants in China are commonly cultivated and naturalized and have been used in traditional Chinese medicine and as food for a long time, including *Impatiens balsamina*, *Mirabilis jalapa*, *Talinum paniculatum* (Jacq.) Gaertn. [72], and *Eryngium foetidum* [73]. Therefore, some exotic plants used by the Thai Hmong may originally relate to traditional knowledge from China before the Hmong migration.

### 3.3. Sources of Hmong’s Knowledge

The Hmong migrated to new homelands and the exotic plants that they now use have arrived along many routes. They brought traditional knowledge with them from their homelands, but also acquired knowledge through acculturation in their new habitats, and in some cases developed entirely new and unique knowledge.

Some species are used by all Hmong, in Thailand, China and other countries where the Hmong settled, suggesting that there has been a long history of acquisition of knowledge related to exotic plants. This use can be said to be part of their traditional knowledge and cultural heritage. In their homeland and wherever they settled, the Hmong used plants to prevent acculturation under their resettlement far away from their homeland, and these plants are actually exotic plants in their new lands. One plant that is widely known by the Hmong is *Bryophyllum pinnatum*, which is used to treat bruises and bone fractures. The Chinese Hmong use it to treat burns, scalds [74], and bone fractures [36], and among the Hmong in Minnesota it is used to treat wounds, chicken pox, fever, stomachache and sores [53]. Additionally, it is used as a symbolic and cultural medicine in Laos [75]. It is also used in folk medicine in tropical Africa, America, India, China, and Australia [76]. Another example is *Canna indica* L., of which the burned rhizome is eaten as an alternative source of starch by many rural people [77]. In addition, the Hmong in Thailand and California use *Canna indica* seeds to treat appendicitis, flatulence, and stomachache [35,41,42,44]. Elsewhere in Southeast Asia, *Canna indica* is used to treat gonorrhea in China, insect bites and swelling in Cambodia, Vietnam, and Laos, and as a diuretic in the Philippines [78]. Thai Hmong used *Impatiens balsamina* and *Mirabilis jalapa* to treat amenorrhoea, dysmenorrhea and for postpartum recovery [37,41,42,45,47]. Laotian refugees in America and California grow *I. balsamina* and use it for hastening childbirth [44,53]. The Hmong in China used the same species for relieving pain, regulating menstruation and treating broken bones [79]. *Mirabilis jalapa* is used to promote delivery of blood clots after birth by the Hmong in Laos [33] and in Minnesota [53]. The Hmong in China used *Mirabilis jalapa* to treat edemas, relieve toxicity and pain, and activate blood flow [79]. Both species are recognized medicines worldwide. *Impatiens balsamina* has been widely used in traditional Chinese medicine to treat rheumatism, difficult labor, and puerperal pain [80]. *Mirabilis jalapa* was used to treat abscesses in India and Java, diabetes in China, infection in Thailand, as a laxative in Europe, and for many other therapeutic purposes in Mexico and Brazil also among indigenous people [81]. Moreover, it is mentioned as a uterine stimulant to hasten childbirth in the USA [82]. *Tradescantia zebrina* is used as a tonic. The Hmong in China use *T. zebrina* to treat gastritis [36]. In Jamaica, Cuba, and Malaysia, *T. zebrina* is highly appreciated as a remedy to improve kidney function, and it is used as nutritional medicine in China [83]. The leaves are decocted, mixed with lemon and drunk as a tonic in Mexico [84]. *Verbena officinalis* is commonly used to treat foot dermatitis and itchy rashes by the Thai Hmong [35,37,40,41,42,45] and to treat indigestion in California [44]. The Chinese Hmong used remedies prepared from *V. officinalis* to treat cold-fever, hepatitis and enteritis [36], to relieve toxicity and pain, regulate menstruation, and to treat broken bones [79]. Moreover, *V. officinalis* is used as medicine in Canada, Italy, Spain and USA [85,86,87] and it has been well-known in the treatment of toxic dermatitis in traditional Chinese medicine for several thousand years [88]. Studies of the pharmacological action of the extract showed that it had significant potential because of its anti-inflammatory effects [89].

Immigrants of the ethnic minorities may come into direct contact with mainstream culture, which may cause acculturation. The knowledge about the many common species has been influenced by knowledge sharing. They were used by the Thai Hmong in the same way as they are used in many other parts of the world. Historically, immigration, commodity exchange, and colonization have been the means of carrying knowledge and have led to the acquisition of plant knowledge by cultural interaction and exchange. Crops and edible plants rose in popularity all over the world. Nowadays, shared information has been diffused by globalization. For instance, *Aloe vera* was used already in the old Mesopotamia, Egypt, and Greece for treatment of the skin, wounds, dermatitis, thermal burn, and sunburn [90]. Today it is frequently used in households and it is currently the most used medicinal plant worldwide [91]. *Aloe vera* is also used by the Hmong who usually grow it in a pot in their home gardens for treating injuries from burns by cutting a leaf and applying the sap to the affected area. Some species used in agricultural extension were introduced in the late 19th Century including *Sechium edule* (Jacq.) Sw. and *Passiflora edulis* [92]. Plant introductions increase interconnectedness in multicultural societies and tend to be universal [93]. Knowledge of exotic plants is also commonly shared in a worldwide context which integrates knowledge for human benefit in cultural globalization. Only a few exotic species used by the Thai Hmong had been the subject of development of new knowledge. Their inclusion here is the first record of their uses by the Hmong and they are, therefore, not broadly used in a worldwide context. They have mostly very specific uses, mostly as medicine and for social purposes.

Some species have been widely used among the Thai Hmong, but are not present in other countries. Perhaps Hmong refugees in Thailand discovered new knowledge that was different from both the original Hmong knowledge, but also different from that of other Hmong migrants. *Chrysanthemum indicum* is native to China with a long history of being planted. Its dried flowers are used for various herbal remedies and they are commonly used as a tea for tonic in China, and also in Thailand [94]. More recently, the Thai Hmong have used its leaves and shoots as a tonic in chicken soup, but this has not been reported for the Hmong from other countries. The first use reported for the Thai Hmong appeared as recently as in 2012 [40,45]; after that, the use of this plant has boomed, and it is now being used by all Hmong in Thailand [52]. *Sambucus canadensis* L. was cultivated in America for its edible fruits, which were used in commercial processors, wineries, jam, and bakeries [95]. To the Thai Hmong, *S. canadensis* does not have an edible fruit, but the leaves are popular and used for treating muscular and skeletal disorders, and injuries by poultice [35,37,42,45]. *Talinum fruticosum* (L.) Juss. is widely used in chicken soup as a tonic and for muscle pain relief [37,40,41,45]. Informants said that they had learned this traditional use from Hmong kinsmen in other villages. They always grow this plant in a pot in their home gardens which shows strong transmission of plant knowledge and it helps in shaping the Hmong identity in Thailand.

Incorporating new exotic plants to generate new Hmong knowledge in Thailand has not happened easily. Changes related to acculturation may be derived from ecological changes induced by an impinging culture [96]. Likewise, Akha refugees in Thailand brought a strong cultural tradition from China with them, but their knowledge has been applied by using a different set of species because of the effect of ecological changes [97]. Resettlement forces newcomers to accommodate to existing new conditions for their life. This involves exotic species that are closely related to taxa that they used with their ancient knowledge to substitute original plants that they easily find in their resettlement area. Thai Hmong still endeavor their cultural heritage. Succulent species in Crassulaceae such as *Bryophyllum*, *Kalanchoe,* and *Sedum* have similar vegetative morphologies and are widely used as medicine by the Hmong. The Thai Hmong say that they can be used for treating bone fractures, injuries, morning sickness, and as food during pregnancy. *Sedum sarmentosum* is native to China and the Chinese Hmong use it to relieve the effects of toxicity, swelling, pain, cough, and to treat broken bones [79]. Laotian Hmong refugees in California used *S. sarmentosum* and *S. spectabile* Boreau to relieve upset stomach and to treat sores [44]; while, in Minnesota, *S. telephium* L. is used for pregnancy and postpartum diet, and to treat bruises and bone fractures [53]. *Talinum fruticosum* is used only by the Thai Hmong, whereas *T. paniculatum* is an exotic plant, which is used around the Hmong culture as a tonic in Thailand [34,40,41,52], Minnesota [53], and to treat incontinence by the Chinese Hmong [36]. Species of *Sedum* and *Talinum* are examples of using closely related taxa as substitutes of the original plants in Hmong culture in different regions. Thai ethnic minorities use plant classifications [98] that correspond closely to scientific taxa [99]. Moreover, they believe that plants are divided into two main groups. The first group is wild herbs that grow naturally in the forest and sometimes include naturalized species. Another group includes the domesticated herbs, which are often exotic species in home gardens [100]. A couple of species may be closely related taxa with the same therapeutic property, but wild plants are thought of as more potent than domesticated plants. Some wild species can be substituted with domesticated exotic plants in pots for easy use. Therefore, plant substitution is a combination of adaptation and cultural recognition. *Sambucus canadensis* was widely used only by the Thai Hmong. A closely related species, *S. javanica* Blume, is native to Thailand and tropical Asia and was used for the same purpose by Thai and Laotian Hmong [37,41,75]. *Sambucus javanica* is thought to be a wild plant whereas *S. canadensis* is a domesticated plant in the folk classification. The Chinese Hmong use *S. adnata* Wall. ex DC. and *S. williamsii* Hance that are both native to China [36]. *Buddleja asiatica* Lour. is a native plant in Thailand and is used as a wild plant for medicine by Thai [35,40,42] and Chinese Hmong [36]. *Buddleja paniculata* Wall. is an exotic plant and a new record as used by the Hmong with the status of domesticated plant. Accordingly, species of *Sambucus* and *Buddleja* are examples of commonly used plant genera in Hmong traditional medicine, but different species are used depending on the region and the status of domesticated plants.

## 4. Materials and Methods

**Figure 2:**
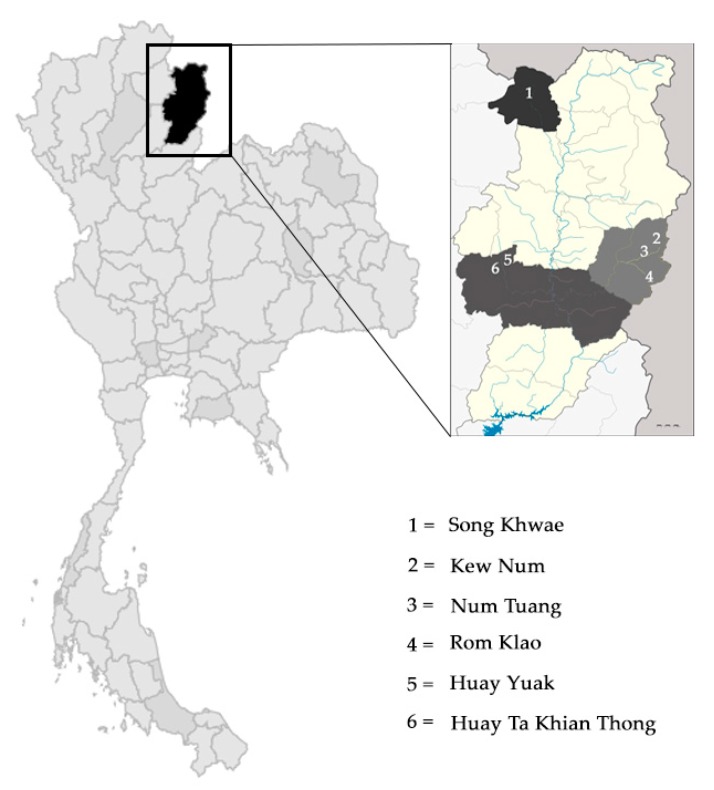
Map of Thailand showing Nan Province and the six Hmong villages studied.

Villagers in six Hmong villages in the Nan province, in northern Thailand (Table 3 and Figure 2), were interviewed about their use of exotic plants. The old people in these villages had migrated from Laos across the border to Nan. We asked local leaders and community members to point to recognized specialists and we selected 16 of them as our key informants [76,101]. Almost all informants were herbalists who used plants in their daily life or shamans who used belief and rituals related to plants in their practice [100,102]. The research protocol was approved by Chiang Mai University Research Ethics Committee with the certificate of approval number COA No. 020/61. Informants were interviewed using semi-structured interviews and field interviews [103]. Questions asked were about the application of plants used, therapeutic properties, and the methods of use. Vouchers were collected of all except seven very common plants for subsequent identification and they were deposited at Queen Sirikit Botanic Garden Herbarium, (QBG), Chiang Mai, Thailand. The plant names were standardized following The Plant List (http://www.theplantlist.org/). Their status as exotic or not was determined following the Thai Plant Names [104]. Use categories followed Cook (1995) for non-medicinal categories [105] and the WHO Classification of Diseases version 11 (ICD-11) for medicinal categories (https://www.who.int/classifications/icd/en/). We cross-checked exotic species with the species described in 16 ethnobotanical studies of Hmong to determine the sources of their knowledge, including Chuakul et al. (2011) [35], Corlett et al. (2003) [44], Culhane-Pera et al. (2004) [100], Committee of Chinese Materia Medica (2005) [79], Lee et al. (2008) [36], Nguanchoo (2014) [37], Nuammee (2011) [38], Pake (1987) [33], Pongsattayapipat (1993) [39], Spring (1989) [53], Srisanga (1994) [34], Srithi (2012) [40], Tichachart (2004) [41], Tovaranonte (1998) [42], Whitney et al. (2014) [75], and Zheng et al. (2013) [74].

## 5. Conclusions

Our research on the traditions of uses of exotic plants by the Thai Hmong demonstrated a deep relationship between exotic species and Hmong culture. The exotic plants provide medicine, food, animal feed, material, and are used for many social purposes. A number of exotic species have a long history of acquisition of the knowledge related to them among the Hmong. The Hmong have used exotic plants since they lived in their original homeland in China and they brought plants into their new habitats when they migrated south, and several species that the Thai Hmong use today are the same plants that were used by the Hmong in China and wherever they have resettled in recent times. This enlightens us about the original traditional knowledge related to the use of exotic plants by the Hmong. However, cultural interaction and globalization have had large effects on shared and commonly used plants worldwide. Acculturation of knowledge and adaption through exotic plants accommodating to existing conditions in the newly colonized lands occurred. Their accommodation is based on inherited knowledge by using closely related taxa as substitutes for the original plants. Our research also shows that the Thai Hmong evolved their current knowledge out of their original plant knowledge through transmission and exchange between Hmong communities. The use of exotic species by the refugee after resettlement is more complicated, and cannot merely be assumed to interfere acculturation and new knowledge because each has different sources, importance, and history.

## Figures and Tables

**Table 1:** Plant families and their numbers of exotic species used in six Hmong villages in Nan province, northern Thailand.

Family	#Species
Asteraceae	8
Amaranthaceae, Solanaceae	5
Euphorbiaceae	4
Amaryllidaceae, Crassulaceae, Cucurbitaceae, Fabaceae	3
Apiaceae, Lamiaceae, Moraceae, Passifloraceae, Talinaceae, Xanthorrhoeaceae	2
Acanthaceae, Adoxaceae, Annonaceae, Araceae, Asparagaceae, Balsaminaceae, Cannaceae, Caricaceae, Commelinaceae, Iridaceae, Malvaceae, Muntingiaceae, Myrtaceae, Nyctaginaceae, Pedaliaceae, Phyllanthaceae, Plantaginaceae, Poaceae, Rhamnaceae, Rubiaceae, Rutaceae, Scrophulariaceae, Verbenaceae	1

**Table 2:** Exotic weeds encountered in six Hmong villages in Nan province, Thailand.

Scientific Name (Varangrat Nguanchoo Voucher Number)	Origin	Global Distribution	Habit	Habitat	Dispersed by
*Amaranthus spinosus* L. (625)	America	Pantropical, widespread in South Africa	Annual	Cultivated beds, open wastelands, margin of streams	Water
*Bidens biternata* (Lour.) Merr. & Sherff (645)	America	Tropical and subtropical Africa, Asia and Australia.	Annual	Orchards, wastelands, re-vegetation areas, forest gaps and margins	Vehicles, water, agricultural produce
*Celosia argentea L.* (652, 923)	Africa	Pantropical, spreading early throughout Asia and Malesia	Annual	Plantations, pastures, open wastelands, and sometime ornamental plants	Wind, water
*Chromolaena odorata* (L.) R.M.King & H.Rob. (624, 970)	America	Tropical and subtropical regions	Perennial	Plantations, rice paddies, pastures, crops, roadsides, wastelands, forest margins, and neglected fields	Wind, water, vehicles, machinery, in clothing, animals, agricultural produce
*Crassocephalum crepidioides* (Benth.) S. Moore. (622)	Africa	Tropical regions	Annual	Coffee and tea plantations, abandoned farmland, wastelands, backyard gardens, shifting cultivation sites	Wind, water
*Mimosa pudica* L. (602, 745, 796)	America	Tropical regions	Perennial	Plantation crops, pastures, lawns, wastelands, along roadsides.	Animal fur, feathers or on clothing
*Passiflora foetida* L. (975)	America	Tropical regions including south-eastern Asia, and many Pacific islands	Annual	Plantation crops, roadsides, wastelands, watercourses, closed forests, open woodlands, and coastal.	Birds and bats with the eaten ripe fruit
*Scoparia dulcis* L. (608, 741, 777)	America	Tropical and subtropical regions	Perennial	Wastelands	Cattle and buffaloes

**Table 3:** Six Hmong villages in Nan province, northern Thailand, where the ethnobotany of exotic plant species was studied.

Village Name	Coordinates (m.a.s.l.)	Distance from Nan City Center (km)	Population#	House- Hold#	Major Economic Crops
**Song Khwae**	19°18’13.12”N 100°44’55.84”E (740)	84	2200	270	Rice, corn, cabbage, bell pepper, lettuce, tomato, lychee, longan, tamarind
**Kew Num**	18°45’52.54”N 101°12’20.37”E (870)	97	289	37	Rice, corn, ginger, black galingale, lychee
**Num Tuang**	18°44’50.37”N 101°11’38.46”E (750)	80	1456	185	Rice, corn, ginger, black galingale, lychee
**Rom Klao**	18°33’31.61”N 101° 2’13.75”E (520)	75	1159	156	Rice, corn, cabbage, bell pepper, cucumber, pea, chili
**Huay Ta Khian Thong**	18°45’45.84”N 100°32’22.28”E (470)	54	349	103	Rice, corn
**Huay Yuak**	18°48’36.72”N 100°31’51.65”E (590)	44	640	98	Rice, corn, ginger, lychee, longan

## Supplementary Materials

The following are available online at https://www.mdpi.com/2223-7747/8/11/500/s1, Table S1: Alphabetical listing of 69 exotic species used by the Hmong in six villages in Nan province in northern Thailand.
