# Supplementary material for: Exotic Plants Used by the Hmong in Thailand
Source: Plants (Basel). 2019 Nov 14;8(11):500. doi: 10.3390/plants8110500 (PMC6918319; doi:10.3390/plants8110500)
Supplement: Supplementary file 1 [file plants-08-00500-s001.pdf]

Table S1. Alphabetical listing of 69 exotic species used by the Hmong in six villages in Nan province in northern Thailand.

For each species the following information is provided: 1. Scientific name and author, family and number of voucher specimens deposited in Herbarium QBG at the Queen Sirikit Botanical Garden in Chiang Mai, Thailand. 2. The geographic origin of the species according to the scientific literature. 3. Use category following Cook (1995) for non-medicinal categories [105] and the WHO Classification for medicinal categories (https://www.who.int/classifications/icd/en/). 4. The application and treatment as observed in the field work for the present study. 5. Preparation and application of the exotic species according to field work for the present study.

**Scientific name** (Family and Varangrat Nguanchoo voucher number, all deposited in the QBG herbarium)	**Geographic origin**	**Use category**	**Application/ Treatment**	**Preparation and application**
Aloe vera (L.) Burm.f. (Xanthorrhoeaceae, no voucher)	Africa	Medicine	Burn, Poisoning due to bites and stings	Gel used for topical use by liniment
Alternanthera bettzickiana (Regel) G.Nicholson (Amaranthaceae, 747, 883)	America	Medicine	Blood tonic, Amenorrhoea, Dysmenorrhea, Urinary tract disease	Decoction for orally drinking
			Bruised	Pound & burn for topical use by poultice
			Fever	Cooked by mixed with egg and steamed for orally eaten
			Tonic	Cooked by boiled with chicken soup for orally eaten
Alternanthera brasiliana (L.) Kuntze. (Amaranthaceae, 633)	America	Medicine	Itchy rash	Decoction for bath
			Sprain (strain)	Pound & burn for topical use by poultice
			Lower abdomen pain caused by retarded ejaculation	Decoction for orally drinking
			Tonic	Cooked by boiled with chicken soup for orally eaten
			Uterine prolapsed	Burn for seat on
		Food	Vegetable	Cooked
	America	Food	Vegetable	Cooked

**Scientific name** (Family and Varangrat Nguanchoo voucher number, all deposited in the QBG herbarium)	**Geographic origin**	**Use category**	**Application/ Treatment**	**Preparation and application**
Amaranthus spinosus L. (Amaranthaceae, 625)		Animal food	Animal food_Pig	Boiled
Anethum graveolens L. (Apiaceae, 676)	Africa to Asia	Medicine	Aphthous ulcer, Chickenpox	Decoction for orally drinking
			Chickenpox	Cooked by boiled with chicken soup for orally eaten
			Fever (children)	Burn for sauna or decoction for orally drinking
		Food	Food additive	Herbs and spices
Annona squamosa L. (Annonaceae, 780)	America	Medicine	Cancer	Decoction for orally drinking
		Food	Fruit	Raw
Artemisia lactiflora Wall. ex DC. (Asteraceae, 683, 714, 916)	Asia (China)	Medicine	Diarrhea	Decoction for orally drinking
			Postpartum recovery (Blood tonic), Tonic	Cooked by boiled with chicken soup for orally eaten
Artemisia vulgaris L. (Asteraceae, 680)	Africa, Europe, Asia to China	Medicine	Headache, Wound	Pound for topical use by poultice
			Fever, Malaria	Cooked by mixed with egg and steamed for orally eaten
			Fever, Relief muscle pain	Decoction for orally drinking
			Liver disorder	Pound & burn for topical use by poultice
Artocarpus heterophyllus Lam. (Moraceae, no voucher)	India to Malaya Peninsula	Medicine	Diarrhea	Decoction for orally drinking
			Lactation stimulant	Cooked by boiled with pork soup for orally eaten
		Food	Fruit	Raw
Ayapana triplinervis (Vahl) R.M.King & H.Rob. (Asteraceae, 721)	America	Medicine	Aphthous ulcer	Raw for chewing
		Food	Vegetable	Raw
Bidens biternata (Lour.) Merr. & Sherff (Asteraceae, 645)	America	Medicine	Aphthous ulcer, Calculus, Chickenpox, Cough, Fever, Gonorrhea, Malaria	Decoction for orally drinking

**Scientific name** (Family and Varangrat Nguanchoo voucher number, all deposited in the QBG herbarium)	**Geographic origin**	**Use category**	**Application/ Treatment**	**Preparation and application**
			Postpartum recovery (food allergic reaction)	Cooked by boiled with chicken soup for orally eaten
Bryophyllum pinnatum (Lam.) Oken (Crassulaceae, 709, 917, 1058)	Africa	Medicine	Abscess, Arthralgia, Bone fractures, Bone pain, Bruised, Flatulence, Inflammation wound	Pound for topical use by poultice
			Blood tonic	Decoction for orally drinking
			Tonic	Cooked by boiled with chicken soup for orally eaten
Buddleja paniculata Wall. (Scrophulariaceae, no voucher)	Asia	Medicine	Abdominal pain during pregnancy	Hot infusion for orally drinking
			Calculus, Leucorrhoea, Female sterility, Peptic ulcer	Decoction for orally drinking
			Headache	Pound for topical use by poultice
Caladium bicolor (Aiton) Vent. (Araceae, 789)	America	Medicine	Peptic ulcer	Cooked by boiled with chicken soup for orally eaten
			Relief muscle pain	Crush for topical use by liniment
			Stomachache	Decoction for orally drinking
		Animal Medicine	Animal Medicine_Pus for buffalo, cow	Pound for topical use by poultice
		Social use	Ritual plant	Treating pain from black magic by pound for liniment
			Sacred plant	Protecting from evil spirit and disease by grown at house
Canna indica L. (Cannaceae, 679, 800, 991, 1054)	America	Medicine	Appendix, Flatulence, Urinary tract disease	Grind or raw for orally eaten
			Appendix, Urinary tract disease, Calculus	Hot infusion for orally drinking

**Scientific name** (Family and Varangrat Nguanchoo voucher number, all deposited in the QBG herbarium)	**Geographic origin**	**Use category**	**Application/ Treatment**	**Preparation and application**
			Blood tonic, Flatulence, Kidney disorder	Decoction for orally drinking
			Tonic	Cooked by boiled with chicken soup for orally eaten
		Food	Carbohydrate source	Burned
Carica papaya L. (Caricaceae, 904)	America	Medicine	Gonorrhoea	Decoction for orally drinking
			Lactation stimulant	Cooked by boiled with chicken soup for orally eaten
			Peptic ulcer	Raw for orally eaten
		Food	Fruit	Raw
		Animal food	Animal food_Pig	Raw
Celosia argentea L. (Amaranthaceae, 652, 923)	Americas	Medicine	Amenorrhoea, Dysmenorrhea, Female fertility	Decoction for orally drinking
			Tonic	Cooked by boiled with chicken soup for orally eaten
Chromolaena odorata (L.) R.M.King & H.Rob. (Asteraceae, 624, 970)	America	Medicine	Burn, Hemostatic, Wound	Pound for topical use by poultice
			Fever	Decoction for orally drinking
Chrysanthemum indicum L. (Asteraceae, 710, 918, 1053)	China and Japan	Medicine	Tonic	Cooked by boiled with chicken soup for orally eaten
Citrus maxima (Burm.) Merr. (Rutaceae, no voucher)	China	Food	Fruit	Raw
Crassocephalum crepidioides (Benth.) S. Moore. (Asteraceae, 622)	Africa	Medicine	Ringworm infection	Crush for topical use by poultice
		Food	Vegetable	Boiled
		Animal food	Pig	Boiled or raw
Crinum × amabile Donn ex Ker Gawl. (Amaryllidaceae, 855)	Asia	Medicine	Abscesses, Bone fractures, Bruised, Sprain (strain)	Pound & burn for topical use by poultice
			Flatulence	Hot infusion for orally drinking
			Peptic ulcer, Stomachache	Decoction for orally drinking

**Scientific name** (Family and Varangrat Nguanchoo voucher number, all deposited in the QBG herbarium)	**Geographic origin**	**Use category**	**Application/ Treatment**	**Preparation and application**
Cucurbita moschata Duchesne (Cucurbitaceae, 717)	America	Food	Vegetable	Cooked
		Animal food	Animal food_Pig	Boiled
Dianella ensifolia (L.) DC. (Xanthorrhoeaceae, no voucher)	Australia, Asia and Africa	Medicine	Amenorrhoea, Cough	Decoction for orally drinking
		Social use	Ritual plant	Used in worship house spirit ritual (Na Jong) by tied with incense and candle and keep a year
Eryngium foetidum L. (Apiaceae, 711)	America	Medicine	Burn	Pound for topical use by poultice
			Headache, Fever	Decoction for orally drinking
		Food	Vegetable	Cooked or raw
Euphorbia tithymaloides L. (Euphorbiaceae, 704, 992, 1047)	America	Medicine	Lactation stimulant, Postpartum recovery (blood tonic)	Cooked by boiled with chicken soup for orally eaten
			Lactation stimulant	Crush for topical use by liniment
Gladiolus hortulanus L.H. Bailey (Iridaceae, 792, 815, 1003)	Africa	Medicine	Bruised, Poisoning due to bite and sting (centipede, scorpion, snake bites)	Pound for topical use by poultice
			Cough	Decoction for orally drinking
		Social use	Ritual plant	Treating pain from black magic by pound for liniment or soul calling by liniment at body
			Sacred plant	Protecting from weapon by putted in pouch
Hibiscus sabdariffa L. (Malvaceae, 621, 821)	Africa	Medicine	Abscesses	Pound for topical use by poultice
			Aphthous ulcer, Blood tonic, Urinary tract disease	Decoction for orally drinking
		Food	Vegetable	Boiled or raw
Hippeastrum x *johnsonii* Bury (Amaryllidaceae, no voucher)	Africa	Medicine	Appendix, Stomachache	Raw for orally eaten
			Cough	Decoction for orally drinking
		Social use	Sacred plant	Protecting from evil spirit by grown at house

**Scientific name** (Family and Varangrat Nguanchoo voucher number, all deposited in the QBG herbarium)	**Geographic origin**	**Use category**	**Application/ Treatment**	**Preparation and application**
Impatiens balsamina L. (Balsaminaceae, 912)	Asia	Medicine	Amenorrhoea, Dysmenorrhea, Female fertility, Fever, Labor induction, Postpartum recovery, Uterine prolapsed	Decoction for orally drinking
			Burn	Pound for topical use by poultice
Iresine herbstii Hook. (Amaranthaceae, 687, 715, 933)	America	Medicine	Amenorrhoea, Dysmenorrhea, Blood tonic	Decoction for orally drinking
			Bruised	Pound for topical use by poultice
			Tonic	Cooked by boiled with chicken soup for orally eaten
Jatropha curcas L. (Euphorbiaceae, 616, 727, 781, 921)	America	Medicine	Aphthous ulcer, Burn, Itchy rash, Wound	latex for topical use by liniment
			Foot dermatitis	Decoction for orally drinking
			Tendon disorders (tendon pain)	Pound for topical use by poultice
		Material	Fence	Living plant grown at house
Jatropha gossypiifolia L. (Euphorbiaceae, 881)	America	Medicine	Amenorrhea	Decoction for orally drinking
			Hemorrhoids, Postpartum recovery (Uterine subinvolution)	Burned for seating on
			Headache, Postpartum recovery (Uterine subinvolution)	Burned for topical use by plaster
		Social use	Sacred plant	Protecting from evil spirit by grown at house near the door
Kalanchoe cf. *ceratophylla* Haw. (Crassulaceae, 685, 958, 993)	India, Asia, china	Medicine	Chest pain	Decoction for orally drinking
			Food allergic reaction, Morning sickness	Cooked by mixed with egg and steamed for orally eaten
			Lactation stimulant, Relief muscle pain, Tonic	Cooked by boiled with chicken soup for orally eaten
	Africa	Medicine	Diphtheria	Hot infusion for orally drinking

**Scientific name** (Family and Varangrat Nguanchoo voucher number, all deposited in the QBG herbarium)	**Geographic origin**	**Use category**	**Application/ Treatment**	**Preparation and application**
Lagenaria siceraria (Molina) Standl. (Cucurbitaceae, 854)			Poisoning due to bite and sting (scorpion bite)	Pound for topical use by poultice
		Material	Dried	Decoration, Water dipper
Manihot esculenta Crantz (Euphorbiaceae, 955)	America	Medicine	Cough	Decoction for orally drinking
		Animal food	Animal food_Pig	Boiled
		Food	Vegetable	Cooked
			Carbohydrate source	Burned or boiled
Mimosa pudica L. (Fabaceae, 602, 745, 796)	America	Medicine	Calculus, Diabetic, Dysmennorhoea	Decoction for orally drinking
			Fever	Pound for topical use by poultice
			Nicotine dependence	Mixed with tobacco for smoking
		Social use	Ritual plant	Soul calling by liniment at body
			Sacred plant	Protecting from evil spirit by putted in pouch
Mirabilis jalapa L. (Nyctaginaceae, 872, 915)	America	Medicine	Amenorrhoea, Postpartum recovery (food allergic reaction)	Decoction for orally drinking
			Bruised, Tonic	Cooked by boiled with chicken soup for orally eaten
			Burn	Pound for topical use by poultice
			Sprain (strain)	Pound & burned for topical use by poultice
Morus alba L. (Moraceae, 932)	China	Medicine	Itchy rash	Decoction for orally drinking
			tonic	Cooked by boiled with pork soup for orally eaten
		Food	Fruit	Raw
Muntingia calabura L. (Muntingiaceae, 731)	America	Food	Fruit	Raw
Nicotiana tabacum L.	America	Medicine	Hemostatic	Pound for topical use by poultice

**Scientific name** (Family and Varangrat Nguanchoo voucher number, all deposited in the QBG herbarium)	**Geographic origin**	**Use category**	**Application/ Treatment**	**Preparation and application**
(Solanaceae, 922, 953)			Poisoning due to bite and sting (mosquito bites)	Crush for topical use by liniment
		Social use	Tobacco	Dried for smoking
Ocimum africanum Lour. (Lamiaceae, 643)	Uncertain	Medicine	Blood tonic	Raw for orally eaten
			Conjunctiva	Grind for drop into eyes
			Liver disorder, Poisoning due to bite and sting (centipede)	Pound for topical use by poultice
		Food	Food additive	Herbs and spices
Passiflora edulis Sims (Passifloraceae, 646)	America	Food	Fruit	Raw
			Vegetable	Cooked
Passiflora foetida L. (Passifloraceae, 975)	America	Food	Fruit	Raw
			Vegetable	Cooked
Phyllanthus acidus (L.) Skeels (Phyllanthaceae, no voucher)	America	Food	Fruit	Raw
Plectranthus scutellarioides (L.) R.Br. (Lamiaceae, 682, 770)	Asia southward to Malesia and Australia	Medicine	Flatulence (infant)	Decoction for orally drinking
			Liver disorder	Crush for topical use by liniment
			Tonic	Cooked by boiled with chicken soup for orally eaten
Psidium guajava L. (Myrtaceae, 609)	America	Medicine	Diarrhea	Decoction for orally drinking or raw for orally eaten
		Food	Fruit	Raw
Psophocarpus tetragonolobus (L.) DC. (Fabaceae, 778)	Asia	Medicine	Chickenpox	Decoction for orally drinking
		Food	Vegetable	Cooked or raw
Sambucus canadensis L. (Adoxaceae, 655, 994, 1042)	America	Medicine	Bruised, Burn, Bone fractures, Fever, Headache, Sprain (strain)	Pound for topical use by poultice
			Sprain (strain)	Decoction for orally drinking
			Tonic	Cooked by boiled with chicken soup for orally eaten

**Scientific name** (Family and Varangrat Nguanchoo voucher number, all deposited in the QBG herbarium)	**Geographic origin**	**Use category**	**Application/ Treatment**	**Preparation and application**
Sanchezia oblonga Ruiz & Pav. (Acanthaceae, 830, 879, 999, 1048)	America	Medicine	Arthralgia, Bone fractures, Bruised, Relief muscle pain, Sprain (strain)	Pound for topical use by poultice
			Bone fractures, Relief muscle pain	Decoction for orally drinking
			Fever	Raw for orally eaten
			Relief muscle pain	Burn for topical use by plaster
			Tonic	Cooked by boiled with chicken soup for orally eaten
Sansevieria trifasciata Prain (Asparagaceae, 615)	Africa	Social use	Sacred plant	Protecting from evil spirit and snake by grown at house
Scoparia dulcis L. (Plantaginaceae, 608, 741, 777)	America	Medicine	Aphthous ulcer	Raw for chewing or crush for topical use by liniment
			Cough	Decoction for orally drinking
Sechium edule (Jacq.) Sw. (Cucurbitaceae, 729)	America	Medicine	Bone fractures	Pound for topical use by poultice
		Food	Vegetable	Cooked
Sedum sarmentosum Bunge (Crassulaceae, 706)	China	Medicine	Female fertility	Decoction for orally drinking
			Food for pregnancy, Morning sickness, Tonic	Cooked by boiled with chicken soup for orally eaten
			Food for pregnancy, Respiration disorder in newborn (cough, suffocate, sputum)	Hot infusion for orally drinking
			Food for pregnancy, Morning sickness	Cooked by mixed with egg and steamed for orally eaten
Senna occidentalis (L.) Link (Fabaceae, 814)	America	Medicine	Flatulence, Stomachache	Decoction for orally drinking
			Food allergic reaction, Peptic ulcer	Hot infusion for orally drinking
Sesamum indicum L. (Pedaliaceae, 880)	Uncertain	Medicine	Cancer	Grind or hot infusion for orally drinking

**Scientific name** (Family and Varangrat Nguanchoo voucher number, all deposited in the QBG herbarium)	**Geographic origin**	**Use category**	**Application/ Treatment**	**Preparation and application**
		Food	Dessert	Raw
Solanum americanum Mill. (Solanaceae, 736, 878)	America	Medicine	Fever, Stomachache	Decoction for orally drinking
		Food	Vegetable	Cooked or raw
Solanum lycopersicum L. (Solanaceae, 612)	America	Food	Vegetable	Cooked or raw
Solanum melongena L. (Solanaceae, 607, 724, 919)	India and Sri Lanka	Medicine	Toothache	Cooked by boiled with chicken soup for orally eaten
		Food	Fruit	Cooked or raw
Solanum torvum Sw. (Solanaceae, 618, 787)	India	Medicine	Liver disorder	Decoction for orally drinking or pound for topical use by poultice
		Food	Fruit	Cooked or raw
Spermacoce ocymoides Burm.f. (Rubiaceae, 935)	Asia	Medicine	Poisoning due to bite and sting (snake bites)	Pound for topical use by poultice
Tagetes erecta L. (Asteraceae, 913)	America	Medicine	Amenorrhoea, Aphthous ulcer, Dysmenorrhea, Female fertility, Postpartum recovery	Decoction for orally drinking
Talinum fruticosum (L.) Juss. (Talinaceae, 751)	America	Medicine	Tonic	Cooked by boiled with chicken soup for orally eaten
Talinum paniculatum (Jacq.) Gaertn. (Talinaceae, 793, 961)	America	Medicine	Postpartum recovery, Tonic	Cooked by boiled with chicken soup for orally eaten
Tradescantia zebrina Bosse (Commelinaceae, 701, 716, 799, 959)	America	Medicine	Amenorrhea, Fever, Relief muscle pain	Decoction for orally drinking
			Food allergic reaction	Hot infusion for orally drinking
			Postpartum recovery, Tonic	Cooked by boiled with chicken soup for orally eaten
			Sprain (strain)	Pound for topical use by poultice
Verbena officinalis L. (Verbenaceae, 605, 794)	Europe	Medicine	Fever	Decoction for orally drinking
			Foot dermatitis, Itchy rash	Crush for topical use by liniment or decoction for bath

**Scientific name** (Family and Varangrat Nguanchoo voucher number, all deposited in the QBG herbarium)	**Geographic origin**	**Use category**	**Application/ Treatment**	**Preparation and application**
			Itchy rash	Pound & mixed with hot oil for topical use by liniment
Zea mays L. (Poaceae, -)	America	Food	Carbohydrate source	Boiled
Zephyranthes carinata Herb. (Amaryllidaceae, 819, 828)	America	Medicine	Diarrhea (children), Epilepsy, Food allergic reaction	Hot infusion for orally drinking
			Food for pregnant	Cooked by mixed with egg and steamed for orally eaten
		Social use	Sacred plant	Protecting from evil spirit by putted in pouch
Ziziphus jujuba Mill. (Rhamnaceae, 730)	Europe to China	Food	Fruit	Raw
